# Gene Variant of *Barrier to Autointegration Factor 2* (*Banf2w*) Is Concordant with Female Determination in Cichlids

**DOI:** 10.3390/ijms22137073

**Published:** 2021-06-30

**Authors:** Arie Yehuda Curzon, Andrey Shirak, Ayana Benet-Perlberg, Alon Naor, Shai Israel Low-Tanne, Haled Sharkawi, Micha Ron, Eyal Seroussi

**Affiliations:** 1 Institute of Animal Science, Agricultural Research Organization, Rishon LeTsiyon 7528809, Israel; arie.curzon@mail.huji.ac.il (A.Y.C.); shiraka@volcani.agri.gov.il (A.S.); micha@agri.huji.ac.il (M.R.); 2 Robert H. Smith Faculty of Agriculture, Food and Environment, Hebrew University of Jerusalem, Rehovot 76100, Israel; 3 Dor Research Station, Division of Fishery and Aquaculture, Hof HaCarmel 30820, Israel; ayanab@moag.gov.il (A.B.-P.); Alonn@moag.gov.il (A.N.); shaiis@moag.gov.il (S.I.L.-T.); haleds@moag.gov.il (H.S.)

**Keywords:** *Oreochromis*, cichlids, *banf2*, sex determination, master key regulator, non-recombining block

## Abstract

*Oreochromis* fishes exhibit variability of sex-determination (SD) genes whose characterization contributes to understanding of the sex differentiation network, and to effective tilapia farming, which requires all-male culture. However, *O. niloticus* (*On*) *amh* is the only master-key regulator (MKR) of SD that has been mapped (XY/XX SD-system on LG23). In *O. aureus* (*Oa*), LG3 controls a WZ/ZZ SD-system that has recently been delimited to 9.2 Mbp, with an embedded interval rich with female-specific variation, harboring two *paics* genes and *banf2*. Developing genetic markers within this interval and using a hybrid *Oa* stock that demonstrates no recombination repression in LG3, we mapped the critical SD region to 235 Kbp on the orthologous *On* physical map (*p* < 1.5 × 10^−26^). DNA-seq assembly and peak-proportion analysis of variation based on Sanger chromatograms allowed the characterization of copy-number variation (CNV) of *banf2*. *Oa* males had three exons capable of encoding 90-amino-acid polypeptides, yet in *Oa* females, we found an extra copy with an 89-amino-acid polypeptide and three non-conservative amino acid substitutions, designated as *banf2w.* CNV analysis suggested the existence of two to five copies of *banf2* in diploidic Cichlidae. Disrupting the Hardy–Weinberg equilibrium (*p* < 4.2 × 10^−3^), *banf2w* was concordant with female determination in *Oa* and in three cichlids with LG3 WZ/ZZ SD-systems (*O. tanganicae,* *O. hornorum* and *Pelmatolapia mariae*). Furthermore, exclusive RNA-seq expression in *Oa* females strengthened the candidacy of *banf2w* as the long-sought LG3 SD MKR. As *banf* genes mediate nuclear assembly, chromatin organization, gene expression and gonad development, *banf2w* may play a fundamental role inducing female nucleus formation that is essential for WZ/ZZ SD.

## 1. Introduction

A master key regulator (MKR) of sex determination (SD) is a gene capable of turning on an alternative gene-regulation program to that maintained at the default homogametic state (XX or ZZ) [1,2,3]. Most mammals have an XX/XY genetic SD system with the same MKR controlling it, i.e., sex-determining region Y (SRY) [4,5]. Similarly, a Z-linked *dmrt1* is the common MKR utilized for the WZ/ZZ system of birds [6,7].

Fish, however, exhibit a remarkable variability of genetic and environmental mechanisms for SD [8] with distinct MKRs in different or even closely related species [9,10]. To date, fewer than a dozen SD MKRs have been reported, with occurrences of XX/XY exceeding WZ/ZZ systems fourfold [8]. According to the dominant gene hypothesis, the heterogametic sex chromosomes (Y or W) often differentiate from their X or Z counterparts by an extra gene copy, which is structurally different from its autosomal ortholog [8]. Alternatively, in a dosage-based hypothesis, two X- or Z-specific genes lead to female or male development, respectively, while a single X- or Z-specific gene is not sufficient to induce such development [11]. Despite the diversity found in fish, most MKRs are factors that have a known function in sexual differentiation and maturation, suggesting that the SD MKR role is performed by a limited number of factors that are part of a conserved cascade or network [3,12]. However, an immune-system-related SD MKR has been found in salmonids [13,14,15]. Nevertheless, extensive evidence still supports a canonical vertebrate gonadal differentiation pathway [8,16]. Thus, SD studies in fish can contribute to a holistic understanding of vertebrate gonadal differentiation mechanisms.

Production of all-male progeny is extremely advantageous for tilapia aquaculture, as it provides a fast and uniform growth rate [17]. Despite environmental and health concerns, currently, mono-sex production is induced by feeding fry with synthetic analogs of androgens [18]. Interspecific artificial fertilization between *Oreochromis* species with different types of SD mechanisms (WZ/ZZ and XX/XY) can yield all-male progeny as a result of the uniform gonosomal profile of the hybrid offspring. This has been reported for crosses of homogametic males (ZZ) of *O. aureus* (*Oa*) or *O. urolepis hornorum* (*Oh*) with homogametic females (XX) of *O. niloticus* (*On*) or *O. mossambicus* (*Om*) [19,20]. Thus, the investigation of the genetic SD mechanism of commercial *Oreochromis* species is highly important for the manipulation of fish breeding towards all-male progeny [21]. In addition to extensive study in the *Oreochromis* clade, SD has also been studied in the wider Cichlidae family, particularly from the African lakes, due to interest in diversification, and as a model for an adaptive radiation evolution process [22,23]. Apart from the MKR, sex can be affected by environment [24], or by additional genetic factors [25]. *Oreochromis* species hybrids are sexually active and are capable of mating amongst themselves and with individuals from the species of origin. Viability and reproducibility of hybrids, and the differences in their MKRs, provide a unique model for studying SD pathways and interactions between different MKRs [26]. Moreover, modulation of meiotic recombination patterns in SD regions from mating of closely related species has been observed and could aid in fine mapping and elucidation of MKRs of SD [27,28,29].

Some mono-factorial SD-systems have been observed in *Oreochromis* species and other cichlids in different independent studies [9,30]. These include XX/XY systems on linkage groups (LGs) 1 [31,32], 7 [23,33] and 23 [26,34,35,36] and WZ/ZZ systems on LGs 3 [31,37,38], 5 [23,39] and 7 [23,33,39]. *Oa* and *Oh* possess the same WZ/ZZ system on LG3, whereas two other species, *On* and *Om*, have different XX/XY systems on LGs 23 and 1, respectively [26]. Like *Oa* and *Oh,* two other cichlid species, *Oreochromis tanganicae (Ot)* and *Pelmatolapia mariae* (*Pm*) have WZ/ZZ systems on LG3 [31,37].

Currently, only in *On* has one of these MKRs, on LG23, been determined. The Y-chromosome (LG23Y) carries an extra copy of *anti-Müllerian hormone* (*amh*), which is different from the regular *amh* gene due to a 5 bp insertion and a 232 bp deletion in exons 6 and 7, respectively [26,34,36]. The WZ/ZZ system on LG3 was initially studied 40 years ago using sex-reversal and gynogenetic techniques and has attracted many studies since [31,37,38,40,41,42,43,44,45,46]. However, the effective MKR has not been found due to the size and complexity of LG3. LG3 is considered to be a mega-chromosome with a high proportion of repetitive elements, recombination repression and a limited feasibility for genetic mapping [41,43]. Indeed, sex chromosomes and sex-determining regions demonstrate a common recombination repression phenomenon during meiosis [47,48]. Comparing male and female linkage map and analyzing FISH staining of the synaptonemal complex, it has been shown that recombination is suppressed in the distal region of the long arms of the largest tilapia chromosome (LG3), which corresponds to the region of delayed pairing in meiosis [49,50]. These observations may be compatible with the two kinds of change affecting recombination rate of evolving sex chromosomes: a gradual reduction of crossover frequencies, due to the spread of genetic modifiers of recombination rates, and chromosome rearrangements such as inversions [51].

A recent study has reduced the SD critical region of *Oa* to 9.2 Mbp [42,43], and, most importantly, it has also identified an embedded critical region with a high female-specific variation density, which harbors only three genes: *banf2*, *paics-1* and *paics-2*. The same study suggests *paics* as a candidate gene for the SD MKR based on chromosomal linkage and the sex-specific expression profile, although mammalian *PAICS* is known to encode the phosphoribosylaminoimidazole carboxylase that participates in the purine biosynthesis without sex-related functions [43].

In the current study, we focused on the genomic region on LG3 noted by Tao et al. for its high density of female-specific patterns of nucleotide variation [43]. To map the SD region, we used a hybrid *Oa* stock with a restored mono-factorial SD locus, which demonstrated a release of recombination repression in the SD region [29]. Based on a conserved trans-species gene variant, which is present exclusively in females, we suggest *banf2w* as a novel MKR for SD in *Oa* and three other cichlid fish that carry the WZ/ZZ SD system on LG3.

## 2. Results

### 2.1. Assembly of Sequence-Read Contigs for Marker Design and Analysis of Association in the Oa SD Region

**Figure 1:**
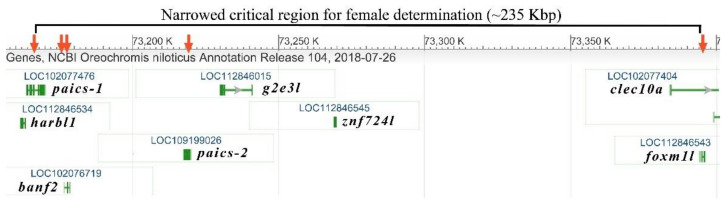
Position of five genetic markers in the SD region of *Oreochromis aureus* delineated on the *O. niloticus* genome map. Genetic markers (red arrows) were developed within *paics-1* (long form), *banf2* (*LOC102076719*), *paics-2* (short form) and *forkhead box protein M1-like* (*foxm1l*). The SD region spanned 235 Kbp between the two external markers.

**Figure 2:**
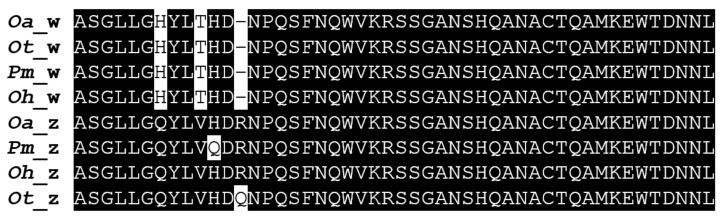
An alignment of the predicted protein domains encoded by the third exons of *banf2w* (nucleotide accession number: OU016046) and *banf2* (nucleotide accession number: OU022051) of *Oreochromis aureus* (*Oa*), *O. tanganicae (Ot*, nucleotide accession numbers: OU016126, OU016104)*, Pelmatolapia mariae* (*Pm*, nucleotide accession numbers: OU017683, OU017684) and *O. urolepis hornorum* (*Oh*, nucleotide accession numbers: OU070172, OU070150) Dashes indicate gaps introduced by the alignment program. Identical amino acid residues in at least three of eight sequences are indicated by a black background. White boxes indicate non-conservative amino acid changes between the proteins.

Based on assembly of DNA-seq *Oa* reads of female and male Sequence-Read Archive (SRA) submissions (SRA accession numbers: ERX2240357 and ERX2240356, respectively) for the genes paics long (*paics-1*) and short (*paics-2a*) forms, *foxm1l* and *banf2*, we designed five genetic markers for studying association of these genes with SD in the *Oa* hybrid stock (Table 1, Figure 1). In *banf2*, examination of multiple SRA submissions (Table S1) revealed coding variation that was concordant with sex (Table S1). Using read-pair information, we assembled distinct haplotypes of the *banf2* genes revealing four haplotypes of *banf2* in an *Oa* female (SRA accession number: ERX2240357) and two haplotypes in an *Oa* male (SRA accession number: ERX2240356). These genes had a typical three-exon structure, including a non-coding first exon, and two coding exons capable of encoding for a 90-amino-acid (aa) polypeptide (Table 2). Two of the female haplotypes (nucleotide accession numbers: OU022051, OU022052) were similar to two male haplotypes (nucleotide accession numbers: OU022053, OU022055), capable of encoding identical 90 aa polypeptides; whereas two female-specific haplotypes had a significant variation that distinguished them from the male haplotypes. One of the female-specific haplotypes had a large deletion in an intronic region, but it did not differ at the protein level from the corresponding male form (nucleotide accession number: OU022054). However, the other haplotype had three aa alterations, including non-conservative aa substitutions, one in exon 2 and two in exon 3, followed by an aa deletion. Capable of encoding 89 aa, this variant may carry out different functions and was designated *banf2w* (nucleotide accession number: OU016046, Table 2, Figure 2).

We designed three additional genetic markers (Table 1): Banf2_del, based on the female-specific haplotype with the intron deletion, and Paics_short and Banf2w, which was assayed using high-resolution melt (HRM) analysis to detect the exon 3 aa alterations of *banf2w* (Figures S1 and S2). These markers were almost completely associated with sex in *Oa* hybrid stock (*n* = 96, a single mismatch, Table 3, Figure S1). However, markers in *paics-1* (Paics_long) and in *foxm1l* (M1_like) showed a much weaker degree of association (Table 3, Table S2), thus mapping the critical SD region to 235 Kbp on the basis of the orthologs *On* genome (Figure 1). The association with SD of the three most significant markers, including Banf2w, fitted the WZ/ZZ SD model (Table 3). Firstly, no W/W individuals were found in a sample of 48 males and 48 females, which significantly deviated from the Hardy–Weinberg equilibrium for inheritance of an autosomal locus (*p* ≤ 4.2 × 10^−3^). Secondly, testing whether homozygotes for this locus are lethal, recessive lethal inheritance of this locus in females was rejected (Table 4).

### 2.2. The Gene Models of Banf2 Locus in Oa Hybrid Stock

**Figure 3:**
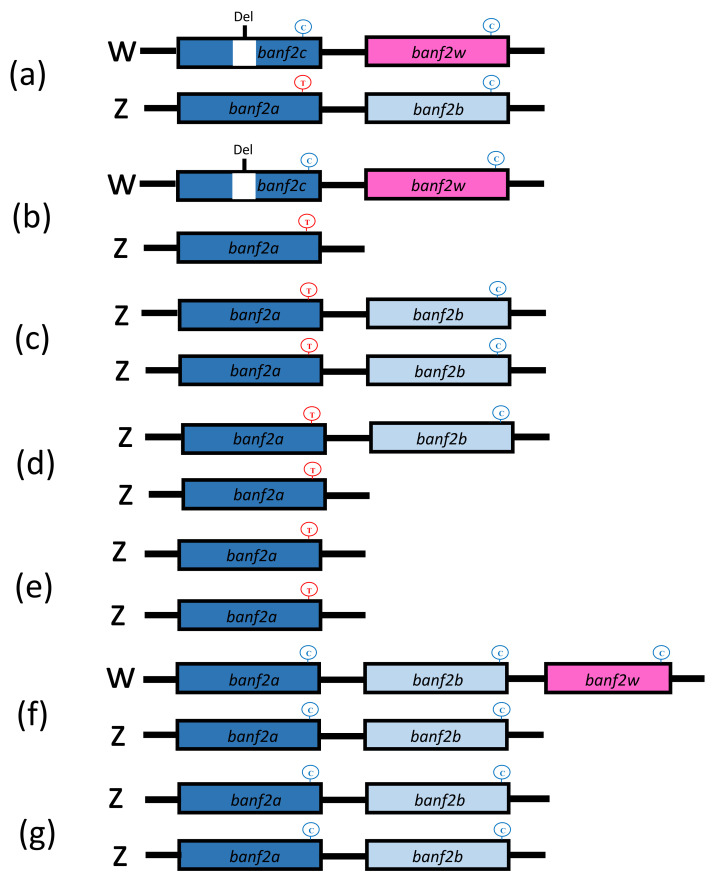
Suggested schematic models depicting the copy number variation of *banf2* genes in the LG3 sex-determining loci of cichlid fish. Models include delineations for *banf2* genes that are not sex-specific (blue boxes) and for the female determination genes (pink boxes). Gene models of *banf2* for females (**a**,**b**) and males (**c**–**e**) demonstrate variability within the *Oreochromis aureus* (*Oa*) hybrid stock and homogeneity of purebred *Oa* females (**b**) and males (**e**). The simplest gene model was observed for the *banf2* locus of both sexes of *O. niloticus* (**e**). A five-copy model represents the gene arrangement predicted for both *O. tanganicae* and *O. urolepis hornorum* females (**f**), and a four-copy model that of males of these species (**g**). “Del” represents a large deletion in *banf2c* intron used as a genetic marker in *Oa*. Shown in circles, an informative C/T/G SNP in exon 3 of *banf2* genes was used to resolve the models in a peak-ratio analysis of Sanger sequencing (also highlighted by red dots in Figure 4).

**Figure 4:**
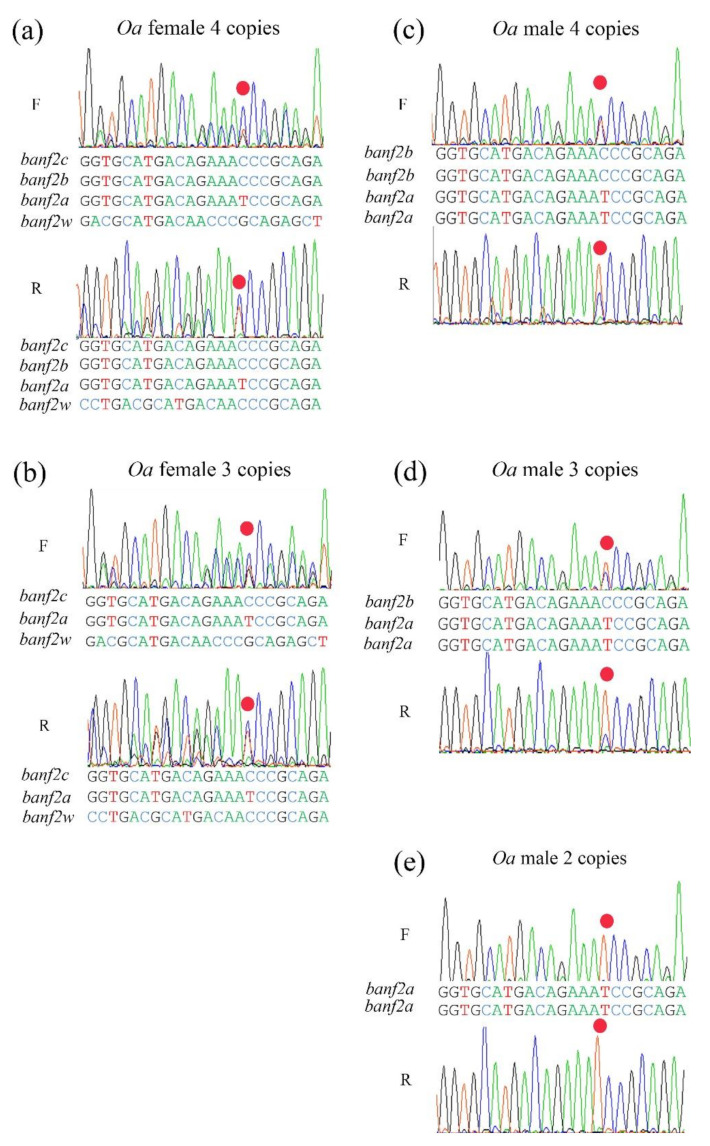
Polymorphism in *Oreochromis aureus* (*Oa*) hybrid stock in Sanger chromatograms of the third exon of *banf2* genes. Chromatograms a-e correspond to models a-e in Figure 4, respectively. Copy proportion analysis was based on both forward (F) and reverse (R) sequencing orientations. The nucleotide sequences below the chromatogram traces were interpreted using Tide software, which, by trace decomposition analysis, estimated that the ratio of *banf2w* was 25% of the signal intensity in some *Oa* females (**a**), and was 33% of this signal in others (**b**), suggesting four and three copies, respectively. The red dots mark an informative position disclosing multiple copies with either C, T or G peaks. For males, this informative site suggested a 2–4-copy model (**c**–**e**). The presence of *banf2a* and *2b* or *2c* was reflected in the observed peak-height ratio between T and C. C was not observed in a homozygous state, and thus it represented a gene duplication that was in tandem with the copy carrying T.

Sequence assemblies of two haplotypes of *banf2* were female-specific, whereas two haplotypes were not, suggesting a model of four *banf2* copies in *Oa* females and four in *Oa* males (Figure 3a,c). This was also supported by the association of the two female-specific haplotypes in the stock with femaleness (Table 3). In addition, sequence assemblies of the *banf2* genes in an artificially produced *Oa* WW individual (SRA accession number: SRX7886422) revealed that it carried only two female-specific *banf2* copies, as was to be expected from the proposed model. Indeed, peak-height analyses of Sanger chromatograms of some female individuals from the hybrid *Oa* stock also supported this model (Figure 4a). For these specimens, *banf2w* represented approximately 25% of the trace signal according to Tide software [52,53], suggesting a 3:1 ratio for *banf2*/*ban2fw* (Figure S3). According to this model, *banf2w* and *banf2c* are located on the W chromosome, while *banf2a* and *banf2b* are situated on the Z chromosome.

To further examine this model, the *banf2* genes were also assembled using additional SRA submissions of male and female *Oa* (SRA accession numbers: SRX8298258, SRX7899544). The male (SRA accession number: SRX8298258) showed two haplotypes as in the sequence assembly of the first male (SRA accession number: ERX2240356). However, unlike the female sequence assembly (SRA accession number: ERX2240357), in this case (SRA accession number: SRX7899544), only three haplotypes were found. Moreover, sequence chromatograms of some of the specimens pointed to additional types of females with presumably three *banf2* copies, in accordance with the decomposition analysis performed by Tide software (Figure 3b and Figure 4b and Figure S3) [52,53]. We were also able to distinguish male types in Sanger sequencing by an informative SNP within exon 3, which allowed deduction of the gene copy proportion by the peak signal ratio of the C/T nucleotides (Figure 4). Three types of males were found in *Oa* hybrid stock, with 2–4 copies of *banf2* (Figure 3c–e). The gene models built based on these specimens suggested that CNV in hybrid *Oa* stock could be resolved by the presence or absence of a *banf2* duplication on the Z chromosome (*banf2b*, Figure 3a,c,d).

### 2.3. The Gene Models of Banf2 Locus in Purebred Oa

**Figure 5:**
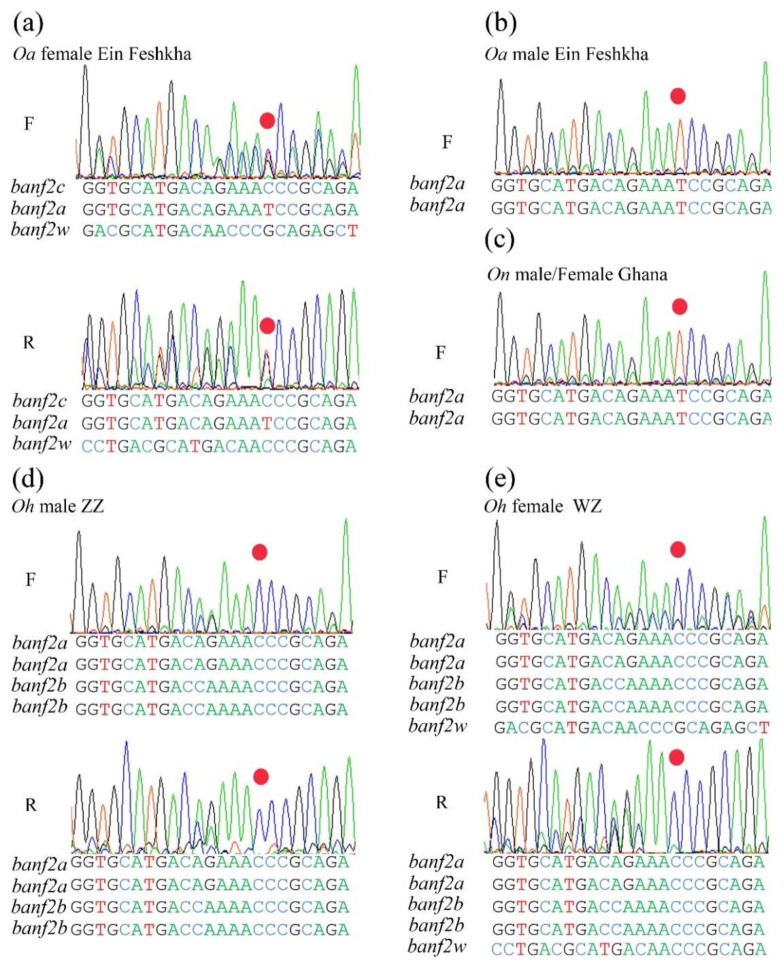
Polymorphism in Sanger chromatograms of third exon of *banf2* genes of three purebred *Oreochromis* species. Typical traces are presented for female (**a**) and male (**b**) of *Oreochromis aureus* (*Oa*); common trace for female and male of *O. niloticus* (*On*) (**c**) and male (**d**) and female (**e**) of *O. urolepis hornorum* (*Oh*). Copy proportion analysis was based on both forward (F) and reverse (R) sequencing orientations. The nucleotide sequences below the chromatogram traces were interpreted using Tide software, which, by trace decomposition analysis, estimated that the *banf2w* was 33% of the signal intensity in *Oa* and was 20% in *Oh*. The red dots mark an informative position disclosing multiple copies in *Oa* females carrying either C, T or G.

Assessment of gene copy proportions by sequence trace decomposition showed that the *banf2w* portion of the signal was stable (~33%, Figure 5a) for sequences of *Oa* females from the Jordan River and the Ein-Feshkha nature reserve, thus suggesting a model of 2:1 for *banf2*/*banf2w* in purebred *Oa* (gene configuration similar to Figure 3b). Accordingly, gene duplication probably occurred on the Z chromosome as a result of hybridization and therefore was not detected in purebred *Oa*. Male sequence chromatograms of purebred specimens also supported this assumption, attested to by the highly informative SNP within exon 3 (Figure 5b).

### 2.4. Banf2w Is Female-Specific in Cichlidae Species with WZ/ZZ SD System on LG3

The *banf2* genes were assembled in two additional cichlid species *Ot* (SRA accession numbers: male: SRX6434465; female: SRX6434463) and Pm (SRA accession numbers: pooled males: SRX3638080, SRX3638081; pooled females: SRX3638084, SRX3638085), in which an orthologous SD locus segregates on LG3 [31,37]. Four haplotypes of *banf2* were assembled in the *Ot* male, whereas in the *Ot* female an additional female-specific copy was found (*banf2w*, nucleotide accession number: OU016126). Comparison of *Ot* and *Oa* suggested a distinct model for the structure of the *banf2* locus (Figure 3f): In the third exon of the *Ot banf2w* gene, aa alterations were similar to that of the *Oa banf2w* gene, although a different aa substitution was observed in exon 2.

It was difficult to assemble and resolve all *banf2* haplotype alleles for *Pm*, as SRA submissions were either of pooled males or females, introducing the additional complexity of polymorphism. Still, the *banf2w* pattern of aa alterations in exon 3 was detected only in the female pool (Figure 2, nucleotide accession number: OU017683). The marker Banf2_del was not informative in *Ot* or *Pm*, as reflected in the sequence assembly results, since the intron deletion is absent in females from these species.

DNA-seq SRA submissions were not available for *Oh*. The genetic markers for *banf2* (Banf2_del) and *banf2w* (Banf2_w) were tested on *Oh* specimens from two families comprising six males and six females from each. Banf2_del was not informative, yet the HRM marker based on the variation of exon 3 in *banf2w* (Table 1) showed full association with femaleness. Moreover, Sanger chromatograms revealed the same conserved aa substitutions in *Oh* banf2w as in *Oa*, *Ot* and *Pm* (Figure 2 and Figure 5). This analysis suggested that both *Oh* and *Ot* had similar gene models because *banf2w*’s signal was approximately 20% of the signal height (Figure 5), fitting a model of 4:1 for *banf2*/*banf2w* ratio (Figure 3f). The synonymous site divergence of *banf2w* reflected the expected cichlid phylogeny placing *Pm* distant from *Oa* (1.1% divergence) and closer to *Ot* and *Oh* (0.6 and 0.0% divergence, respectively).

### 2.5. Competitive Expression of Banf2w and Other Banf2 Genes in Oa Females

**Figure 6:**
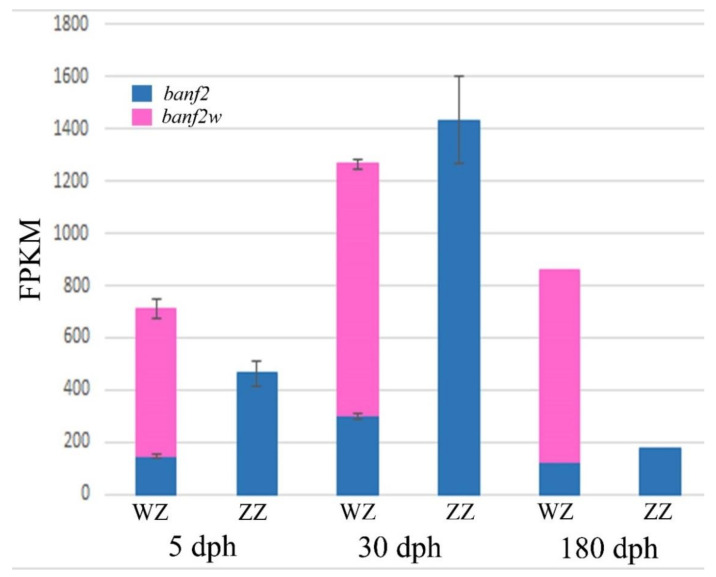
Expression in units of fragments per kilobase million (FPKM) of *banf2w* and other *banf2* genes in *Oreochromis aureus* gonads of males (ZZ) and females (WZ) at three ages: 5, 30 and 90 days post-hatching (dph). The minimal threshold for expression detection was set to three reads.

*Banf2w* and *banf2* expression were compared between *Oa* SRA submissions of RNA-seq analyses of gonad transcriptomes of females and males (SRA accession numbers: SRX7906433 to SRX7906446, *n* = 14). *Banf2w* was exclusively present in female gonads in all developmental stages including 5, 30 and 180 days post-hatching (dph). It had a relatively higher expression value in females when compared to other *banf2* genes (*p ≤* 1 × 10^−5^) and reached its peak expression at 30 dph (Figure 6). *Banf2* gene expression was generally higher in males (*p ≤* 1.5 × 10^−2^, ZZ), specifically at 5 (*p ≤* 2.6 × 10^−3^) and 30 dph (*p ≤* 2.1 × 10^−3^), when compared to females (Figure 6). It is noteworthy that expression of *banf2* increased threefold in males between 5 and 30 dph with similar pattern of expression in both sexes peaking at 30 dph. Also of note is that the expression of *banf2* decreased in males and converged to values that were similar to those of female expression at 180 dph; however, significance was not tested at this stage due to lack of replicates. The total *banf2* FPKM value of gene expression (Z and W) was slightly higher in females at 5 dph (*p ≤* 1.2 × 10^−3^) but was insignificantly enhanced at 30 dph (Figure 6). Interestingly the search for expression of *banf2w* in *Ot* adult ovary (SRA accession numbers: SRX6445742, SRX6445762, SRX6445765) did not detect any reads, whereas *banf2* was abundant.

### 2.6. The Paics Gene Candidates for SD

At *Oa* LG3 SD locus, Tao et al. noted only *paics-1* and *paics-2* [43]. Based on the current genome build (assembly accession number: GCF_013358895) of a “ZZ” individual, two *paics-1* genes were annotated as *ade2-like* genes (*LOC116316740*, *LOC120434429*). An additional member of the gene family has been placed between these genes (*LOC116321116)*. In *On*, the *LOC116321116* orthologous gene is mapped to LG23 as *ppat* and is adjacent to another *paics* gene, which is orthologous to *Oa* LG23 *paics* (Gene ID: 116331053). However, in the *foxml1* gene, the genetic marker M1_like was associated with LG3 SD in our *Oa* stock (Table 3); it was mapped in this current *Oa* genome build to LG23. These observations suggest that in the current *Oa* genome build, LGs 3 and 23 might have been disordered; alternatively, the structure of LG3 in the hybrid stock substantially differs from that of the ZZ individual. Moreover, the ZZ-based *Oa* LG3 build is missing the *paics-2a* gene and thus differs from LG3 of *Oa* W genome. Based on assembly of DNA-seq reads from a WW individual (SRA accession number: SRX7886422), we partially inferred the *paics-2a* gene sequence (nucleotide accession OU234059). In exon 3, which was capable of encoding 65 aa, we encountered coding variation including six aa substitutions compared to the *paics-1* paralogs (*LOC116316740*, *LOC120434429*), for which the third exon encoded identical polypeptides. A BLASTP search against the non-redundant GenBank dataset (NR) showed that *paics-2a* encoded a novel Paics unique to *Oa* females. To examine expression of *paics-2a*, we followed the procedure described for *banf2w*, testing 14 gonad transcriptomes using a 32 bp probe “CGAGTCTCAAGACCAGATCACAGCTGGGAACA” that represented the unique variation in exon3 of *paics-2a*. In ovaries, at five dph, the average expression was 1.2 FPKM, and it peaked at 180 dph reaching 71.03 FPKM. No expression was detected in testes. However, TBLASTN search against *Ot* and *Pm* SRA data indicated that *paics-2a* was specific to *Oa* and not conserved in females of these other species. We also failed to detect the 5’ *paics-2a* nucleotide sequence (Paics_short) in *Ot* and *Pm*. Thus, with no trans-species conservation *paics-2a* is a less likely candidate for an SD MKR.

## 3. Discussion

In this study, we describe *banf2* genes of four cichlid species (*Oa*, *Ot*, *Oh* and *Pm*). These species have a known WZ/ZZ SD system, which has been mapped to genomic regions orthologous to the *On* LG3. For all four species, we identified a female-specific copy of *banf2*, designated *banf2w*. Conserved among the four species, a female-specific aa deletion and non-conservative aa substitutions in exon 3 of *banf2w* strengthened the candidacy of *banf2w* as a novel MKR of SD. Moreover, highlighting the uniqueness of this finding, a female-specific allele of *banf2* with a large deletion in the second intron (*banf2c*, Figure 3) showed the same degree of association with sex in *Oa* as *banf2w* but was not observed in the other three species (Figure 3).

The region containing *banf2* has previously been suggested as a candidate region for SD based on its high female-specific variation density in *Oa* [43]. This reported region includes only three genes: *banf2*, *paics-1* and *paics-2* [43]. As a follow up study, we characterized the proposed SD critical region harboring these three genes in an *Oa* hybrid stock, mapping it to 235 Kbp, based on the ortholog physical *On* genome (genome assembly *O_niloticus*_UMD_NMBU). Previously, *paics* has been proposed as a candidate MKR for SD in *Oa* based on differential expression between sexes. However, we did not find any conserved coding variation across different species for this gene to support its candidacy. Moreover, for the genetic marker designed in *paics-1* (Paics_long), association with sex was weakened by multiple segregants. The marker in *paics-2a* (Paics_short) showed the same degree of association with sex as *banf2w*. However, without sex-related functions [43] or conservation across species, the *paics-2a* gene is unlikely to be the femaleness determiner. Hybrids were shown here to be a useful tool for characterization of the SD region. The actual mechanism of how hybridization of species with different SD systems induces meiotic recombination within the non-recombining blocks of SD regions is not clear. However, it is probably related to chromosomal sequence and structural differences between the mating species. MKRs on cichlid LGs 1, 5 and 7, are still unidentified. The discovery of *amh* as MKR *for SD* in *On* [54,55,56] has promoted the identification of the role of the orthologous *amh* genes in SD of distant fish species [57,58,59,60]. Similar implications for research are expected for *banf2* genes.

Additional support for the candidacy of the *banf* gene family to affect SD is the observation that one of the four well-narrowed chromosomal regions of QTLs for spontaneous female-to-male sex reversal harbors *banf1* in rainbow trout *(Oncorhynchus mykiss*) [61]. Therefore, it is important to sequence the *banf1* genes in these males to locate genetic mutations that possibly underlie this spontaneous sex reversal.

Investigating the expression profile of *banf2w*, we found exclusive expression in *Oa* females, indicating that this gene was functional and had a sex-specific expression profile. In adult *Ot* female specimens, however, *banf2w* expression was not observed, which may be explained by a limited time window of expression of this gene in *Ot*. Similarly, a male-specific MKR for SD, *fshry,* has been reported for *Mugil cephalus* with no observed expression in males (40 dph to adulthood) [8,62]. *Sry* in mammalian males is constitutively expressed from early embryonic stages to adulthood in different tissues, implicating its role as a housekeeping gene [63]. An interesting open question for future research is why an MKR is constitutively expressed in some organisms and not in others.

In *Oa* females (30–180 dph), transcription of *banf2w* was higher than other *banf2* genes. However, *banf2* gene expression was low in comparison with male expression for all stages. These results were significant at 5 and 30 dph, while at 180 dph there were no replicates to allow significance testing. Taken together, the results indicate that *banf2* expression observed in male larvae (1–30 dph and maybe older) was replaced in females by *banf2w*. Expression of *banf2* in males and *banf2w* in females reaches a peak at 30 dph, which is similar to that of the SD *amh* in *On* males [36]. These peaks of expression, observed for different MKRs, coincide with the end of the time window in which hormonal and temperature masculinization can be induced [24].

As with most MKRs of SD, *banf2w* has a known function that associates it with SD. Mammalian *banf2* is highly and almost exclusively expressed in testis [64] and is upregulated during spermatogenesis [65]. Banf2 is thought to be a regulator of Banf1 [64]. Interaction between Banf1 and Banf2 occurs through formation of homo- and hetero-dimers, which is especially important in testis [64]. *Banf1* is a repressor of *foxl2*’s pro-apoptotic activity [66,67] and is involved with gonad development [64,68]. Thus, acting in chromatin assembly, the nuclear-lamina-associated Banf1 protein may indirectly impact granulosa cell differentiation and apoptosis [66,67]. Bound to emerin complexes in the nuclear envelope [69], Banf1 may repress *foxl2* by mediating its relocation to the nuclear periphery [67]. *Amh* and *foxl2* are central genes regulating testis and ovary development, respectively [2,70]. This is driven by their pro-apoptotic activity, which results in morphological changes of the bipotential gonad [71,72]. Thus, Banf2w may have a dominant effect by preventing the gene silencing function of Banf1. Shutdown of this function would promote *foxl2′*s apoptotic activity and consequently testis suppression and ovarian development. Overall, we postulate that *banf2w* has the potential for programing gene expression in cells, causing a switch to a femaleness state via modulation of the chromatin structure, thereby inducing female nucleus formation.

The suggested models of *banf2* gene structures in *Oa*, *Ot* and *Oh* are based on short sequence-read assemblies, and analysis of peak ratios at polymorphic sites in Sanger chromatograms. Demonstrating that a combination of the two methods is a useful tool in characterizing the CNV of complex loci governing SD, we showed that *banf2w* is an extra copy of *banf2* in females. Presumably, *banf2w* evolved by gene duplication and speciation. Indeed, *On* with an XX/XY SD system on LG23 did not carry a duplication of *banf2*. Similar structural variation for SD loci is frequent, indicating a typical gene formation pathway. In the closely related *On* species, an extra copy of *amh* in males governs sex (*amhΔy*); this is located <40 Kbp apart from the regular *amh* copy and is under the same promoter. However, in the present work, we did not determine the physical map of the *Oa* W chromosome containing *banf2w* and *banf2c* genes. Understanding these gene orientations and promoter positions, and comparing them with other similar cases, is important for deciphering mechanisms in which duplications evolve into specialized SD MKRs.

In the *Oa* hybrid stock, only partial association with sex was observed for the UNH168 marker that has been previously reported to be associated with sex in *Oa* and *Oh* [38,44]. However, the genetic markers Banf2_del, Paics_short and Banf2w showed almost complete association with sex in this stock. Through marker-assisted selection for these three SD loci, nearly all-male progeny in *On* × *Oa* crosses was obtained, thus demonstrating the mono-factorial SD on LG3. Only a single *Oa* male was found with a WZ SD genotype, without any male-determination alleles for the genetic markers for SD on LGs 1 and 23. This discrepancy may be explained by environmental sex reversal, involvement of other genetic factors or appearance of non-parental genotype in *banf2* locus mediated by aberrant crossing-over [29] or unequal crossing over [73,74].

Both *On* female and male (Figure 5c), and *Oa* male (Figure 5b) gene models displayed a single copy of *banf2* (*banf2a*). However, some *Oa* hybrid males were heterozygous or homozygous for an extra gene copy on the Z chromosome (*banf2b*). This additional copy produced sequence chromatograms similar to those of the *banf2a* copy, except for the informative T to C substitution (Figure 4). We did not find *banf2b* in our Egypt and Ghana *On* strains, yet it has been detected in GenBank (*On* Japanese and BYL78 strains, SRA accession numbers: SRX7899813, SRX726489). Hence, this gene variant may have originated from hybridization with an *On* strain. In contrast to Middle East and North Africa species *Oa* and *On*, two *Oreochromis* species of Central and South Africa, *Ot* and *Oh*, did have *banf2b* (Figure 3f,g). Thus, another possibility is that a contaminator of the *Oa* hybrid stock was either *Oh* or *Om,* both of which are common in aquaculture. It is important to note that the CNV in *banf2* locus in the hybrid stock was not associated with SD and suggested that female sex is driven by the actual presence of *banf2w* and not by a *banf2* dosage effect. Thus, unlike in medaka where simple duplication created a new female determiner in line with the dosage-based hypothesis [75], *banf2w’s* role in SD better fits the dominant gene hypothesis. This should be further explored by functional validation using advanced methods, such as CRISPR/Cas9, TALEN, or antisense RNA [36,60,76,77].

## 4. Materials and Methods

### 4.1. Fish

One-year-old females and males (48 of each) were randomly selected from an *Oa* hybrid stock of the Dor research station [29] and were used for analysis of genetic markers in the LG3 SD region for association with sex. Two additional male and female individuals from this stock have been previously sampled for Illumina HiSeq 2000 paired end sequencing (BioSample accession numbers: SAMEA104362031 and SAMEA104362030). *Oh* specimens from two families were received from a Fish Aquaculture Station in Brazil [78]. Purebred *Oa* specimens from local natural resources (Jordan River and Ein-Feshkha Reserve) and *On* specimens from different introduced strains (Ghana, Swansea and Canada) have been previously described [26].

### 4.2. DNA Extraction, PCR Amplification, Agarose Separation and Sanger Sequencing

A sample of the caudal fin (100–200 mg) preserved in ethanol was used for DNA extraction using a commercial kit (MasterPure DNA Purification, Madison, WI, USA). PCR was performed using relevant primers that were designed using Primer3 [79] (Table 1) and the Bio-X-ACT™ Long kit (Bioline Ltd., London, UK) according to the manufacturer’s instructions under the following conditions: 36 cycles for 30 s at 94 °C, 30 s at 59–63 °C and 30–50 s at 72 °C. Thereafter, the PCR products were examined for genotyping on the basis of size in a 1–2% agarose gel stained with ethidium bromide. Following excision from the gel, Sanger sequencing was conducted from both directions of the purified products (Montage Gel Extraction, Millipore, Bedford, MA, USA).

### 4.3. Marker Development and Search for Coding Polymorphism in O. aureus

Genetic markers were developed in the region on LG3 that contains *paics* and *banf2* and has the highest density of sex-specific polymorphism in *Oa* [43]. For the sequence assembly of four genes (*paics-1*, *paics-2*, *foxm1l* and *banf2*) and identification of potential functional polymorphism, two previously published male and female *Oa* genomic-DNA libraries (SRA accession numbers: ERX2240356 and ERX2240357, respectively) were used (Figure 1, Table 1). Five developed markers (paics_long, paics short, M1-like, Banf2_del and Banf2w) with DNA length or SNP polymorphism were tested for association with sex by AGE or by a HRM analyses. Genotyping by HRM was conducted using a Real-Time PCR instrument (ECO, Illumina, California, SD, USA) and the qPCRBIO HRM Mix (PCR Biosystems, London, UK). In addition, UNH168, a microsatellite marker found to be highly associated with LG3 in several studies [38,44] was also examined. PCR for UNH168 used dye-labeled forward and unlabeled reverse primers (Table 1). The amplified products were separated on an ABI3130 DNA sequencer and sized using the GeneMapper software v. 4.0 (Applied Biosystems Ltd., Foster City, CA, USA) with GeneScan-500 LIZ size standard (Applied Biosystems) [80].

### 4.4. Copy Number Estimation with Sanger and Construction Genetic Models

Copy numbers were estimated taking into account coverage of next generation sequencing (NGS) reads, and copy number proportion was measured by Sanger chromatograms [52]. Proportions of copies were estimated using Tide software [52,53]. Each chromatogram analysis was performed using template DNA of at least six individuals with the same sex phenotype and genotypes. Such individuals yielded similar chromatograms of which a typical one was selected for further analysis and presentation.

### 4.5. Sequence Alignments

Protein sequences were aligned with ClustalW (http://clustalw.genome.jp, accessed on 29 June 2021), using default settings and Gonnet weight matrix. Nucleotide sequences were aligned using the same tool and the DNA option. The graphical image of the multiple alignment was made using BoxShade (https://www.ch.embnet.org/software/BOX_form.html, accessed on 29 June 2021). To calculate synonymohttps://embnet.vital-it.ch/software/BOX_form.htmlus (accessed on 29 June 2021) site divergence of *banf2w*, the *banf2w* nucleotide sequences were aligned and substitutions were counted within positions 2261–2438 (nucleotide accession number: OU016046). An indel was counted as a single event.

### 4.6. Expression Analysis

FPKM values were used for comparing expression values of different haplotypes. Fourteen RNA-seq libraries (SRA accession numbers: SRX7906433 to SRX7906446) were aligned against 32 bp probes of each haplotype, using the SRA Nucleotide BLAST [81] with parameters that force complete fit (32bp word). The numbers of aligned hits for each haplotype were then recorded for calculation of the FPKM.

### 4.7. Statistics

The JMP© statistical package (Pro 13, SAS Institute, Cary, NC, USA) was used for conducting Fisher’s exact chi-squared and Pearson chi-squared tests, which were applied for association study of genetic polymorphism and sex and for goodness of fit of inheritance models, respectively. In addition, one-way analysis of variance (ANOVA) was used for comparing differences in expression data of different developmental stages and haplotypes.

## Figures and Tables

**Table 1:** Polymerase chain reaction (PCR) primers for amplicons used as genetic markers within genes at the critical region for sex determination on LG3.

Marker	Primers		Assay	Accession No.
UNH168	F	FAM-TAAGAAGGTTAGAAAGAAAGTG	PAGE ^1^	G12320
	R	TATATAATAATTTCCTAAACGGC		
Paics_long	F	GCGGGAGACTAGCTGCAATA	AGE ^2^	NC_031967
	R	TTGCAGCACATGGACAGTAG		
Paics_short	F	TCCTCACGTGGAAATCAATG	AGE	OU234059
	R	CCACCAGCTGGAAAATCTGT		
Banf2_del	F	CTACTCTGGGGAGGGAGCTG	AGE	OU023198
	R	TGACTGTTTGCTCCACTGCT		
Banf2w	F	CTCTGGCCTCCTCGGTCA	HRM ^3^	OU019478
	R	TGACTGTTTGCTCCACTGCT		
M1_like	F	GGCTAATATTTTGTTGTGTGTAGGG	AGE	NC_031967 XM_025906134
	R	AGGAACAACTGCTCTTCAGGA		
Banf2_EX3	F	CCAATCTTCTTGTTCCTGACC	Sanger sequencing	OU019478
	R	GAGGTGCCTCTCAGGTAAAGG		

^1^ Polyacrylamide-gel electrophoresis (PAGE) of fluorescent products. ^2^ Agarose-gel electrophoresis (AGE) with ethidium bromide staining. ^3^ High-resolution melt analysis.

**Table 2:** Genomic organization of the *Oreochromis aureus banf2w* gene.

Intron ^1^	Exon		Intron	
	No.	Size (bp)		Size (bp)
GTTAAAGTTCTA	1	119	AAACAG**gt**tcgtttgc	1002
ttcttctc**ag**G**ATG**TC	2	127	AGCAAG**gt**gcacaaaa	668
gtgttttc**ag**GCCTCT	3	231	CTG**TGA**GGCCCCGCCCCCTTTACCT GAGAGGCACCTCTCAGCTGATTAT GTGCTGAGCATG**AATAAA** ACATA**AATAAA**GTTTGTGTGCCGGAG	

^1^ Intron and exon sequences are written in lowercase and uppercase letters, respectively. The first and last two bases of the introns are presented in bold type (**gt** and **ag** for donor and acceptor splice sites, respectively). The initiation and stop codons and the putative polyadenylation signals (**ATG**, **TAG**, **AATAA**) are shown in bold and underlined. Starting from the transcription initiation site, the genomic and transcript sizes of the *banf2w* gene were 2147 and 477 bp, respectively.

**Table 3:** Markers and their association with sex in *Oreochromis aureus* hybrid stock (*n* = 96).

Marker	Position (Kbp)	Genotypes	Females: Males	*p*-Value
UNH168	64,515	N/N ^1^	5:12	6 × 10^−12^
		N/W	22:2	
		N/Z	8:29	
		Z/W	13:0	
		Z/Z	0:5	
Paics_long (*Paics-1*)	73,159	F/F ^2^	27:14	3.9 × 10^−3^
		F/S	21:29	
		S/S	0:5	
Banf2_del	73,177	F/S ^2^	48:1	**1.5 × 10^−26^**
		S/S	0:47	
Banf2_w	73,178	W/Z	48:1	**1.5 × 10^−26^**
		Z/Z	0:47	
Paics_short (*Paics-2*)	73,217	P ^3^	48:1	**1.5 × 10^−26^**
		A	0:47	
M1_like	73,394	F/F ^2^	4:5	6.8 × 10^−3^
		F/S	25:10	
		S/S	19:33	

^1^ N—*On* originating alleles, W—*Oa* W allele, Z—*Oa* Z allele. ^2^ F—fast-migrating fragment, S—slow-migrating fragment. ^3^ Paics_short is a dominant marker, P—fragment is present, A—fragment is absent. The bold is the most significant probabilities.

**Table 4:** Goodness of fit of inheritance models with the observed distribution of Z/W based on *banf2* genotypes in females of *Oa* stock (*n* = 48).

	ZZ	WZ	WW	
	Observed Distribution			
	0	48	0	
Inheritance Models ^1^	Expected Distribution			^2^ *p*
Autosomal locus ^3^	12	24	12	3.8 × 10^−11^
Lethal WW homozygote ^4^	16	32	0	6.4 × 10^−5^
Female determining W ^5^	0	48	0	1

^1^ H_0_—there is no difference between observed and expected distributions based on the inheritance model with allele frequencies *p* (Z) = 0.5 and *q* (W) = 0.5. ^2^ Pearson chi-squared test. ^3^ Hardy–Weinberg equilibrium: *p*^2^, 2*pq*, *q*^2^. ^4^ Elimination of *q*^2^: *p*^2^/(1 − *q*^2^), 2*pq*/(1 − *q*^2^), 0. ^5^ Elimination of *q*^2^ and *p*^2^: 0, 1, 0.

## Data Availability

Data are contained within this article’s supplementary material. Nucleotide sequence data for *Oa*, *Oh*, *Ot* and *Pm* were deposited in ENA under project accession number PRJEB44866.

## Supplementary Materials

The following are available online at https://www.mdpi.com/article/10.3390/ijms22137073/s1.
