# Supplementary material for: Gene Variant of Barrier to Autointegration Factor 2 (Banf2w) Is Concordant with Female Determination in Cichlids
Source: Int J Mol Sci. 2021 Jun 30;22(13):7073. doi: 10.3390/ijms22137073 (PMC8268354; doi:10.3390/ijms22137073)
Supplement: Supplementary file 1 [file ijms-22-07073-s001.zip › FigureS1-S3.pdf]

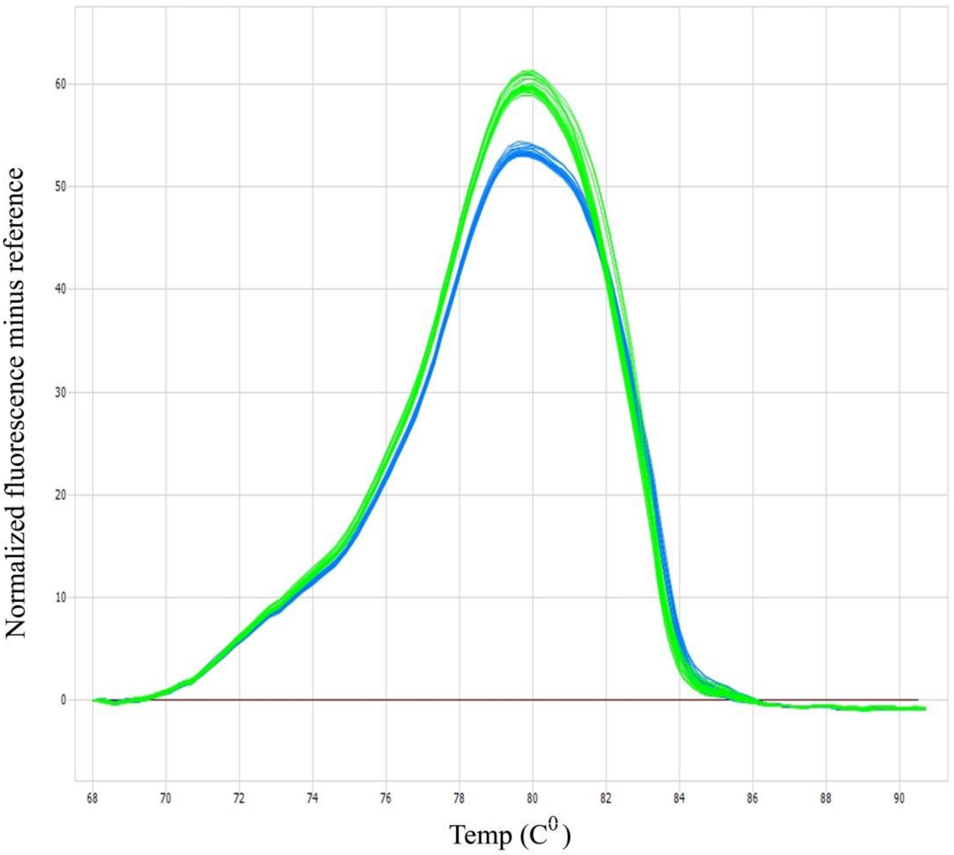

Figure S1: An example of the high-resolution melt analysis of *Oreochromis aureus* males (green, n=20) and females (blue, n=20) using PCR primers flanking exon 3 of *banf2* and *banf2w* genes. The fluorescence was normalized against a control blank sample.

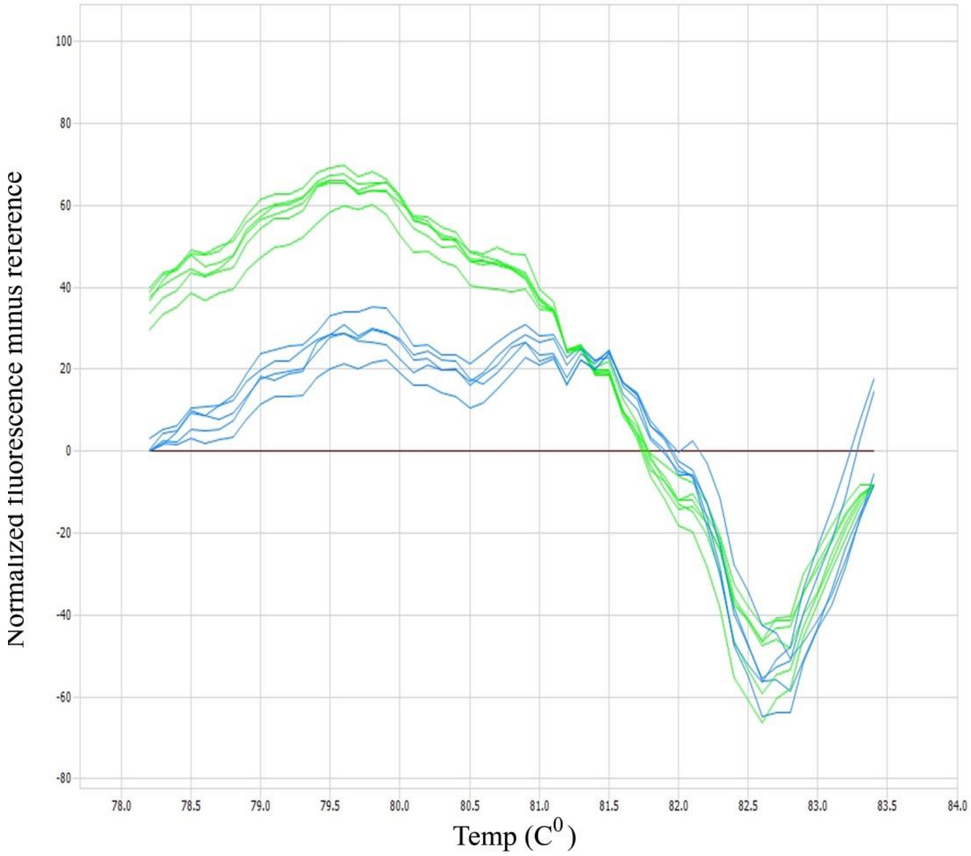

Figure S2: An example of the high-resolution melt analysis of *Oreochromis urolepis hornorum* males (green, n=6) and females (blue, n=5) using PCR primers flanking exon 3 of *banf2* and *banf2w* genes. The fluorescence was normalized against a control blank sample.

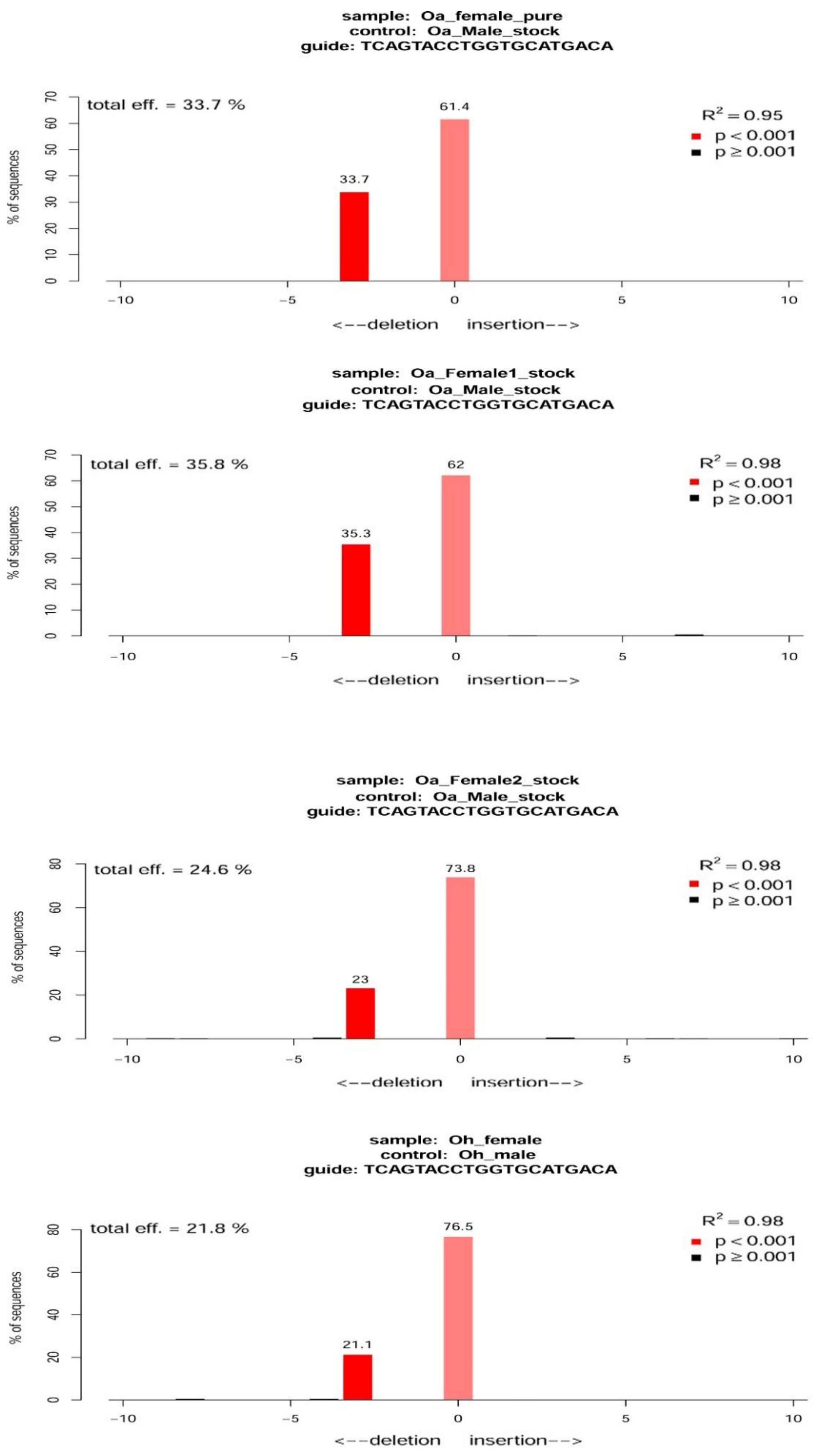

Figure S3. Peak-height analyses of copy-number variation in Sanger chromatograms of *Oreochromis banf2* genes. Percentage of *banf2* (orange) and *banf2w* (red) sequences are displayed including: a purebred *O. aureus* female from Ein-Feskha nature reserve, two *O. aureus* females (1 and 2) from a hybrid stock and an *O. urolepis hornorum* female. Analyses were performed using the Tide software (Brinkman et al, Nucl. Acids Res. 2014) and were based on decomposition of double peaks caused by co-sequencing long-form *banf2* genes with *banf2w*, which had a 3bp deletion in exon 3. Males of both species, which do not carry *banf2w*, were used as the control sequences.
