# Supplementary material for: Gene Variant of Barrier to Autointegration Factor 2 (Banf2w) Is Concordant with Female Determination in Cichlids
Source: Int J Mol Sci. 2021 Jun 30;22(13):7073. doi: 10.3390/ijms22137073 (PMC8268354; doi:10.3390/ijms22137073)
Supplement: Supplementary file 1 [file ijms-22-07073-s001.zip › TableS1.pdf]

Table S1. Detection of *Oreochromis aureus* (*Oa*) *banf2w* in GenBank SRA submissions.

Oa strain	Sex	SRA submissions	*Banf2w* present[1] (+) or absent[2] (-)
Gb001	Male	SRX8298258	-
ARO male	Male	ERX2240356	-
Vienna	Male pool	SRX7899812	-
Maryland	Male pool	SRX2436283	-
Maryland	Female pool	SRX2436282	+
ARO	Female	ERX2240357	+
Vienna	WW superfemale	SRX7886422	+
Guangdong	Female	SRX7899539<br>SRX7899540<br>SRX7899541<br>SRX7899542<br>SRX7899543	+
Chongqing	Female	SRX7899544<br>SRX7899545<br>SRX7899546<br>SRX7899547<br>SRX7899548	+
Guangzhou[3]	Male	SRX8063999<br>SRX8064000<br>SRX8064001	+
Guangzhou	Female	SRX8063996<br>SRX8063997<br>SRX8063998	-

[1] Verified by BLAST searches against the SRA submissions using a 32 bp *banf2w* probe "GCCTCTGGCCTCCTCGGTCACTACCTGACGCA".

[2] The detection threshold was set to 3 sequence reads.

[3] Contradicting to all other observations, in the Guangzhou strain *banf2w* was only detected in males. Analysis of assosiation for a female allele for a genetic marker which was reported by the submitter of this strain sequences indicated an erroneous switch in gender annotations in these SRA submissions. This was verified by BLAST searches of a 32 bp probe "CCATGAAGCACATGACAAACAGCAGGGTGAGA". The probe was planned by sequence assembly of Marker 2 in Table. S4 of the submitter's paper. (Tao et al, Mol. Ecol. Resour. 2021)
